# Baduanjin sequential therapy’s effects on quality of life and cardiac function in post-cardiac surgery heart disease patients: A systematic review

**DOI:** 10.1097/MD.0000000000046855

**Published:** 2026-01-02

**Authors:** Xuezhi Liu, Xingxing Hao, Wenhui Zhang, Fan Zhang, Hui Liu

**Affiliations:** a Department of Cardiovascular Surgery, Linfen Central Hospital, Linfen, Shanxi, China.

**Keywords:** Baduanjin exercise, cardiac function, cardiac surgery, cardiovascular heart disease, meta-analysis, quality of life

## Abstract

**Background:**

This study aimed to objectively evaluate the effects of Baduanjin exercise on the quality of life and cardiac function in patients with cardiovascular heart disease after cardiac surgery.

**Methods:**

Pubmed, Embase, Cochrane, Web of science, Chinese National Knowledge Infrastructure, Wanfang and Sinomed were searched from the date of their inception until March 5th, 2024 using medical subject headings terms and keywords. The primary outcomes were the quality of life and cardiac function. The quality of life was assessed using Short Form-36, the Seattle Angina Questionnaire, 6-min walk test and adverse events. The cardiac function was evaluated using the 6-min walking test. For statistical analysis, standardized mean difference or odds ratio and 95% confidence intervals were calculated using Stata 14.0.

**Results:**

Baduanjin exercise demonstrated significant enhancements in quality of life across all Short Form-36 subitems. Meta-analyses revealed improvements in Seattle Angina Questionnaire scores related to physical limitation and stable angina pectoris. The incidence of adverse events decreased with Baduanjin exercise, and cardiac function, as indicated by left ventricular ejection fraction, showed significant improvement.

**Conclusions:**

Baduanjin exercise is a safe, feasible, and acceptable intervention that can improve the quality of life and cardiac function in patients with cardiovascular heart disease after cardiac surgery. However, more studies with rigorous research designs are needed to assist in the rehabilitation of such patients.

## 1. Introduction

With the aging of the global population, cardiovascular heart disease (CHD) has become one of the most prevalent illnesses worldwide, and its incidence continues to rise each year. In China, CHD has become the second leading cause of death.^[1]^ The number of CHD cases in China is increasing rapidly and is expected to reach 23 million by 2030.^[2]^ Although the elective revascularization procedures for coronary artery disease, including percutaneous coronary intervention (PCI) and coronary artery bypass grafting (CABG), are widely used and highly effective treatments for CHD, many patients continue to experience angina, psychological distress, and reduced cardiac function after cardiac surgery.^[3–5]^ Within the first year after a myocardial infarction, more than half of survivors die and nearly 50% require hospitalization.^[6]^

It has been reported that appropriate exercise regimens can rapidly induce a cardiac phenotype capable of resisting the effects of chronic ischemia-reperfusion injury on the myocardium.^[7]^ Furthermore, exercise promotes microvascular regeneration and helps regulate autonomic nervous system balance.^[8,9]^ Therefore, in addition to pharmaceutical therapy, exercise is considered an important component of CHD management, as it reduces cardiovascular diseases, lowers the incidence of angina and cardiac events, and improves patients’ quality of life.^[10]^ However, traditional exercise-based rehabilitation is often lengthy, expensive, and difficult to implement, posing particular challenges for patients with CHD in low-income ettings.^[11]^ Therefore, a simple, accessible, and affordable exercise approach is needed as a complementary therapy for this population.

Baduanjin exercise, also known as the Eight-Section Brocades, is a traditional form of Chinese Qigong that is simple to learn and practice, requiring no equipment or space restrictions.^[12]^ Unlike traditional aerobic or resistance training, Baduanjin focuses on coordinated postures, fluid movements, mindful breathing and techniques to cultivate qi (vital energy based on the theory of traditional Chinese medicine), thereby promoting both physical and mental well-being.^[13]^ An increasing body of evidence has demonstrated the health benefits of Baduanjin in various populations, including improvements in balance, flexibility, muscle strength, physical fitness, and mood disorders such as depression.^[14–16]^ In addition, Baduanjin has been reported to enhance left ventricular ejection fraction (LVEF), cardiac output, and stroke volume, while lowering resting myocardial oxygen demand in older adults, indicating its suitability for individuals with cardiovascular conditions.^[17]^

Recently, several studies have explored the impact of Baduanjin on quality of life and cardiac function in patients with CHD following PCI.^[18,19]^ However, the literature still lacks a comprehensive systematic review assessing its benefits for individuals undergoing cardiac surgery. Therefore, this systematic review and meta-analysis aimed to synthesize existing evidence and provide an objective evaluation of the therapeutic effect of Baduanjin exercise postoperative quality of life and cardiac function in patients with CHD.

## 2. Materials and methods

### 2.1. Study registration

This systematic review and meta-analysis was conducted in accordance with the Preferred Reporting Items for Systematic Review and Meta-analysis 2020 guidelines.^[20]^

### 2.2. Search strategy

Seven electronic databases (PubMed, Embase, Cochrane Library, Web of Science, Chinese National Knowledge Infrastructure, Wanfang, and Sinomed) were searched. The following terms were used in different combinations: Baduanjin, Tai Chi, Taiji, Qigong, and coronary heart disease. Detailed retrieval strategy was listed in Table S1, Supplemental Digital Content, https://links.lww.com/MD/R61. The bibliographies of included studies for additional references were manually identified.

### 2.3. Eligibility criteria

The studies were included if they met the following criteria:

(1) Types of participants: the participants were patients with CHD after PCI or CABG. No restriction on their age, race and country.
(2) Types of studies: Random controlled trials or controlled clinical trials of Baduanjin for cardiac function or the quality of life in patients with CHD or CABG after PCI were included.
(3) Types of intervention/control: The experimental group included patients treated with Baduanjin as the primary intervention. The control group included patients who received Western medicine or Chinese herbal medicine or walking exercise, with the exception of Baduanjin.
(4) Types of outcome measures: Two outcomes for assessing the treatment effects of Baduanjin exercise were assessed in CHD patients after cardiac surgery. The quality of life was assessed using the Short Form-36 (SF-36), the Seattle Angina Questionnaire (SAQ), 6-minute walk test (6MWT) and adverse events. Domain scores for the SF-36 included physical function, role limitation physical, bodily pain, vitality, role limitation emotional, mental health, social function, and general health perception.^[21]^ According to the domain scores, physical health component summary and mental health component summary scores were calculated. All domain and summary scores ranged from 0 to 100 with higher scores indicating better quality of life. The SAQ had 5 dimensions related to angina: the exertional capacity scale, anginal stability scale, anginal frequency scale, treatment satisfaction scale and the disease perception scale.^[22]^ The 6MWT is a widely available and well-tolerated test which has been used to assess the functional and exercise capacities in patients with CHD. Major adverse cardiovascular events included nonfatal myocardial infarction, target vessel blood rehabilitation, recurrent angina, congestive heart failure and severe arrhythmia, etc. The cardiac function was assessed using LVEF, which was obtained through echocardiography.

The studies were excluded if they met the following criteria: duplicate publications; conference abstracts, reviews, clinical trial protocol, and animal studies, were excluded; and literature with no-reported outcomes.

### 2.4. Assessment of risk of bias

The risk of bias for all selected experimental studies was assessed by 2 independent reviewers. Cochrane Risk of Bias assessment tool (Rob2) was used for assessing risk of bias in randomized trials and the Risk of Bias in Non-randomized studies of Intervention (ROBINS-I) was used for non-randomized trials.^[23,24]^ Disagreements were resolved through discussion or consultation with a third reviewer.

### 2.5. Data extraction

Primary data were extracted using a standardized scale (the Cochrane Effective Practice and Organization of Care Review Group data collection checklist), including first author names, publication year, country of publication, sample size, patient characteristics (age and sex), the type of cardiac surgery, the follow-up time, the interventions taken for experimental group and control group, and outcome of measurement. One author performed data extraction, which was independently reviewed by 2 other authors. Disagreements were resolved through group consultation.

### 2.6. Statistical analysis

Meta-analysis was performed using STATA 14.0 (Stata Corporation, College Station). Each outcome was calculated using the mean difference (MD) or standardized mean and 95% confidence interval (CI) between the intervention and control groups. *I*^2^ values (<25%, low; 25–75%, medium; >75%, high) and the chi-squared test (*P* ≥ .1, good homogeneity; *P* < .1, significant heterogeneity) were used to evaluate the heterogeneity of the data. A fixed-effects model was used when heterogeneity was low; otherwise, a random-effects model was applied. Forest plots were used to display the results graphically. Publication bias was assessed using funnel plots and Egger test for asymmetry. Subgroup analyses were performed to explore the possible source of heterogeneity. Sensitivity analysis (leave-one-out method) was performed to assess the influence of any particular study on the pooled estimate. Statistical significance was set at *P* < .05.

## 3. Results

### 3.1. Literature Search

**Figure 1.**
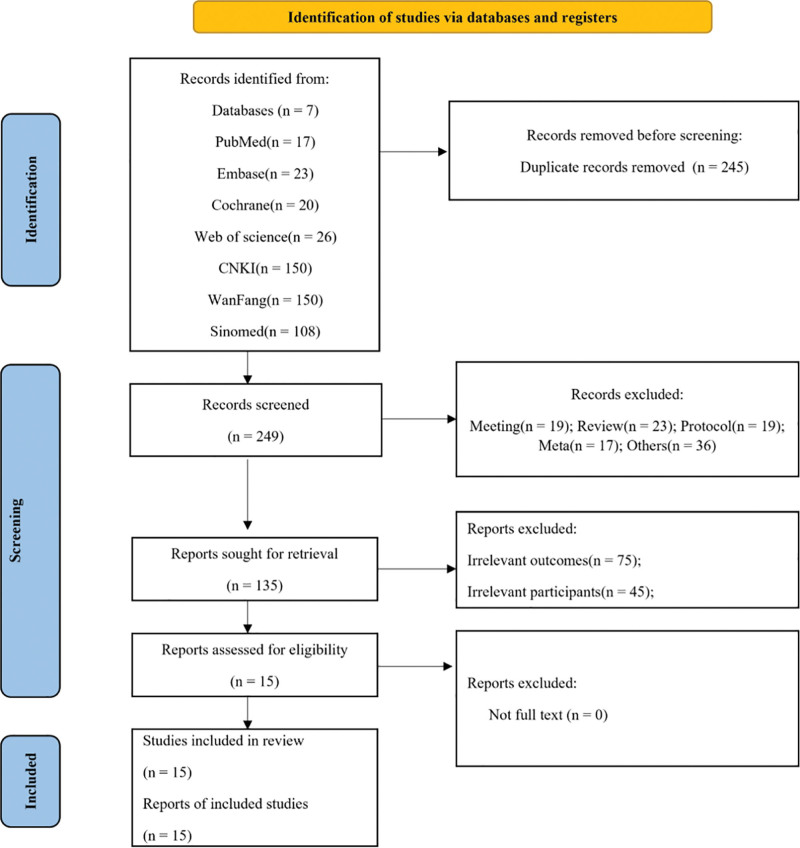
The flowchart of this study.

A total of 494 relevant records were identified through the 7 search databases. After removing 245 duplicates based on title and author, 249 articles remained. A total of 114 articles were excluded because of ineligible article type, such as review, conference paper, protocol, meta-analysis or others. An additional 120 full-text articles were excluded after screened for their titles and abstracts for irrelevant outcomes or irrelevant participants. Finally, 15 articles were included.^[7,25–38]^ Among them, one study was published in English and fourteen in Chinese. Figure 1 illustrates the detailed flow of the study selection process.

### 3.2. Study characteristics

The main characteristics of the included studies are presented in Table 1. These studies were conducted in China and published between 2012 and 2023. Among them, one was published in English, while the remaining fourteen appeared in Chinese. The sample size in this study ranged from 43 to 245, with a total of 1384 participants (697 in the intervention group and 687 in the control group). The age of participants ranged from 45.55 to 65.69 years. Intervention duration varied from 1 week to 6 months: four studies lasted 6 months, two lasted 5 months, five lasted 3 months, one lasted 2 months, two lasted 1 month, and one lasted 1 week. In the included trials, Baduanjin exercise was in comparison with conventional therapy (drug therapy or rehabilitation therapy). Outcomes reported included SF-36 (7 studies), SAQ (6 studies), 6MWT (5 studies), adverse events (5 studies), and LVEF (6 studies).

**Table 1:** Characteristics of included studies.

Study	Country	Study design	Patients	Surgery	Sample sizes (experiment/control group)	Age	Female%	Intervention of experiment group	Intervention of control group	Follow-up time	Outcomes
Lin et al^[28]^	China	RCT	Coronary syndrome	Coronary artery bypass grafting	30/30	65.69	23.33	Baduanjin exercise	Medical gymnastics, walking, power cycling and other sports	5 mo	SAQ
Gu et al^[33]^	China	RCT	Coronary syndrome	PCI	50/50	59.37	26	Baduanjin exercise	Drug therapy	3 mo	SAQ
Jing 2019	China	RCT	Coronary syndrome	PCI	55/55	59.32	50	Baduanjin exercise	Drug therapy	5 mo	LVEF, SF-36
Wang et al^[30,37]^	China	RCT	Coronary syndrome	PCI	30/30	59.81	35	Baduanjin exercise	Walking exercise	6 mo	SAQ, SF-36
Chen et al^[7]^	China	RCT	AMI	PCI	43/39	60.7	28.05	Baduanjin exercise	Blood revascularization + drug therapy	6 mo	SF-36, AEs
Yan and Shuqin^[25]^	China	RCT	Acute coronary syndrome	PCI	32/32	57.14	6.25	modified Baduanjin exercise	Walking exercise	3 mo	6MWT
Chen et al^[38]^	China	RCT	Coronary syndrome	PCI	30/30	62.84	30	Baduanjin exercise	Conventional rehabilitation treatment	6 mo	SF-36
Gong et al^[27]^	China	RCT	Coronary heart disease combined with type 2 diabetes	PCI	30/30	62.49	38.33	Baduanjin exercise	Drug therapy	3 mo	SF-36
Li et al^[36]^	China	RCT	Coronary syndrome	Coronary artery bypass grafting	124/121	60.03	20.41	Baduanjin exercise	Conventional rehabilitation treatment	1 wk	6MWT, LVEF
Zhang et al^[32]^	China	RCT	Coronary syndrome	PCI	40/40	63	42.5	Baduanjin exercise	Conventional rehabilitation treatment	3 mo	LVEF, SAQ, 6MWT
Liang et al^[31]^	China	RCT	AMI	PCI	23/20	45.55	43.75	Baduanjin exercise	Drug therapy	6 mo	SF-36, AEs
Rong 2022	China	CCT	Coronary syndrome	PCI	80/80	61.56	46.875	Baduanjin exercise	Conventional rehabilitation treatment	1 mo	6MWT, SAQ
Wang et al^[23,26]^	China	RCT	Coronary syndrome	PCI	40/40	52.25	31.25	Baduanjin exercise	Conventional rehabilitation treatment	2 mo	6MWT, AEs, LVEF
Wei and Meng^[34]^	China	CCT	AMI	PCI	30/30	53.54	40	Baduanjin exercise	Conventional rehabilitation treatment	1 mo	LVEF, AEs
Zhang et al^[29]^	China	RCT	AMI	PCI	60/60	53.1	50	Baduanjin exercise	Drug therapy	3 mo	SF-36, SAQ, AEs, LVEF

6MWT = 6-minute walk test, CCT = controlled clinical trial, LVEF = left ventricular ejection fraction, PCI = percutaneous coronary intervention, RCT = random controlled trial, SAQ = Seattle Angina Questionnaire, SF-36 = Short Form-36.

### 3.3. Risk of bias

Thirteen randomized controlled trials were assessed for risk of bias using RoB2. Some concerns were present for bias in measurement of the outcome for Li (2021), whereas all other studies had low concerns (Fig. S1, Supplemental Digital Content, https://links.lww.com/MD/R61). Overall, all studies had a low risk of biases (Fig. S2, Supplemental Digital Content, https://links.lww.com/MD/R61). The risk of bias in other 2 controlled clinical trials were evaluated using ROBINS-I. All 2 controlled clinical trials had the low risk of biases (Figs. S3 and S4, Supplemental Digital Content, https://links.lww.com/MD/R61).

### 3.4. Meta-analysis for outcomes measures

#### 3.4.1. Short Form-36

Seven studies evaluated patients’ quality of life using the SF-36 scale. As this instrument consists of 8 domains (general health, physical functioning, role-physical, role-emotional, social functioning, bodily pain, vitality, and mental health), we performed separate meta-analyses for each subscale. The detailed results for all studies are summarized in Table 2.

**Table 2:** Meta-analysis on the subitems of SF-36.

The content SF-36	Heterogeneity test		Effect model	Meta-analysis	
	*P*	*I^2^*		MD (95% CI)	*P*
Bodily pain	<.001	99.3%	Random effect model	9.60 (0.58 to 18.63)	.037
General health	<.001	83.0%	Random effect model	6.23 (0.58 to 11.88)	.031
Mental health	.015	64.5%	Random effect model	5.16 (1.26 to 9.05)	.009
Physical functioning	.211	30.0%	Fixed effect model	6.78 (4.57 to 8.99)	<.001
Role emotional	<.001	84.9%	Random effect model	11.16 (1.60 to 20.72)	.022
Role physical	.013	68.3%	Random effect model	11.20 (3.41 to 18.99)	.005
Social function	.025	61.2%	Random effect model	9.70 (4.34 to 15.06)	<.001
Vitality	.006	72.2%	Random effect model	10.20 (5.45 to 14.94)	<.001

CIs = confidence intervals, MD = mean difference, SF-36 = Short Form-36.

From the pooled analysis of these 7 studies, we found that Baduanjin exercise could improve the score of SF-36 in all 8 subitems (all *P* < .05).

A funnel plot showed an asymmetric graph (Fig. S3, Supplemental Digital Content, https://links.lww.com/MD/R61). The values of Begg test and Egger test suggested a publication bias for the SF-36 in physical functioning (Begg test *P* = .039 and Egger test *P* = .003). Furthermore, the sensitivity analysis revealed no significant change in the overall effects after excluding any of the studies (Fig. S4, Supplemental Digital Content, https://links.lww.com/MD/R61).

#### 3.4.2. Seattle Angina Questionnaire

Five studies evaluated patients’ quality of life using the SAQ. Meta-analyses were performed for its key domains, including angina frequency, disease perception, physical limitation, stability of angina, and treatment satisfaction. The corresponding results for each study are presented in Table 3. From the pooled analysis of these 5 studies, we found that Baduanjin exercise could improve the score of SF-36 in physical limitation and stable angina pectoris (MD = 8.04, 95% CI 1.56 to 14.49, *P* = .014, *I*^2^ = 98.1%; MD = 10.28, 95% CI 2.64 to 17.91, *P* = .008, *I*^2^ = 98.8%).

**Table 3:** Meta-analysis on the subitems of SAQ.

The content SF-36	Heterogeneity test		Effect model	Meta-analysis	
	*P*	*I^2^*		MD (95% CI)	*P*
Attack of angina pectoris	<.001	99.3%	Random effect model	8.36 (−0.85 to 17.57)	.075
Degree of disease awareness	<.001	99.4%	Random effect model	11.61 (−1.36 to 24.58)	.079
Physical limitation	<.001	98.1%	Random effect model	8.04 (1.56 to 14.49)	.014
Stable angina pectoris	<.001	98.8%	Random effect model	10.28 (2.64 to 17.91)	.008
Treatment satisfaction	<.001	98.5%	Random effect model	6.37 (−2.73 to 15.48)	.171

CIs = confidence intervals, MD = mean difference, SAQ = Seattle Angina Questionnaire.

The funnel plot demonstrated a degree of asymmetry (Fig. S5, Supplemental Digital Content, https://links.lww.com/MD/R61). However, Begg and Egger tests indicated no significant publication bias for the SAQ outcomes (both *P* > .05). In addition, sensitivity analyses showed that removing individual studies did not materially alter the pooled effect estimates (Fig. S6, Supplemental Digital Content, https://links.lww.com/MD/R61).

#### 3.4.3. Six-minute walk test

**Figure 2.**
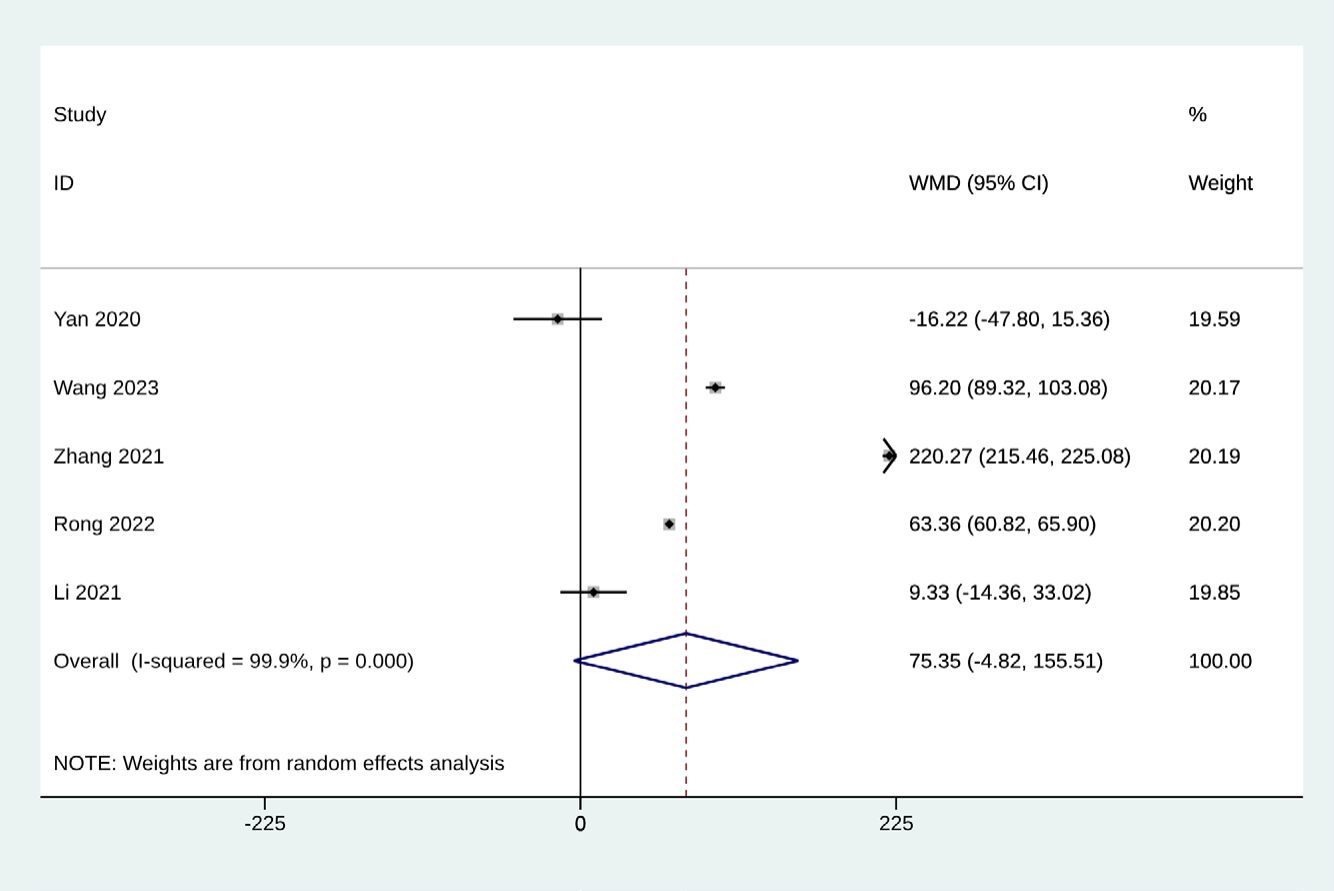
The effect of Baduanjin exercise on 6MWT score. 6MWT = 6-minute walk test, CI = confidence interval.

Five studies reported the effect of Baduanjin exercise on 6MWT score. We did not find that Baduanjin exercise could increase 6MWT score from the meta-analysis (MD = 75.35, 95% CI −4.82 to 155.51, *P* = .065, *I*^2^ = 99.9%, Fig. 2). No significant publication bias was observed, as indicated by the funnel plot (Fig. S7, Supplemental Digital Content, https://links.lww.com/MD/R61) and Egger test results (*P* = .827). The sensitivity analysis confirmed the stability of the results (Fig. S8, Supplemental Digital Content, https://links.lww.com/MD/R61).

#### 3.4.4. Adverse events

**Figure 3.**
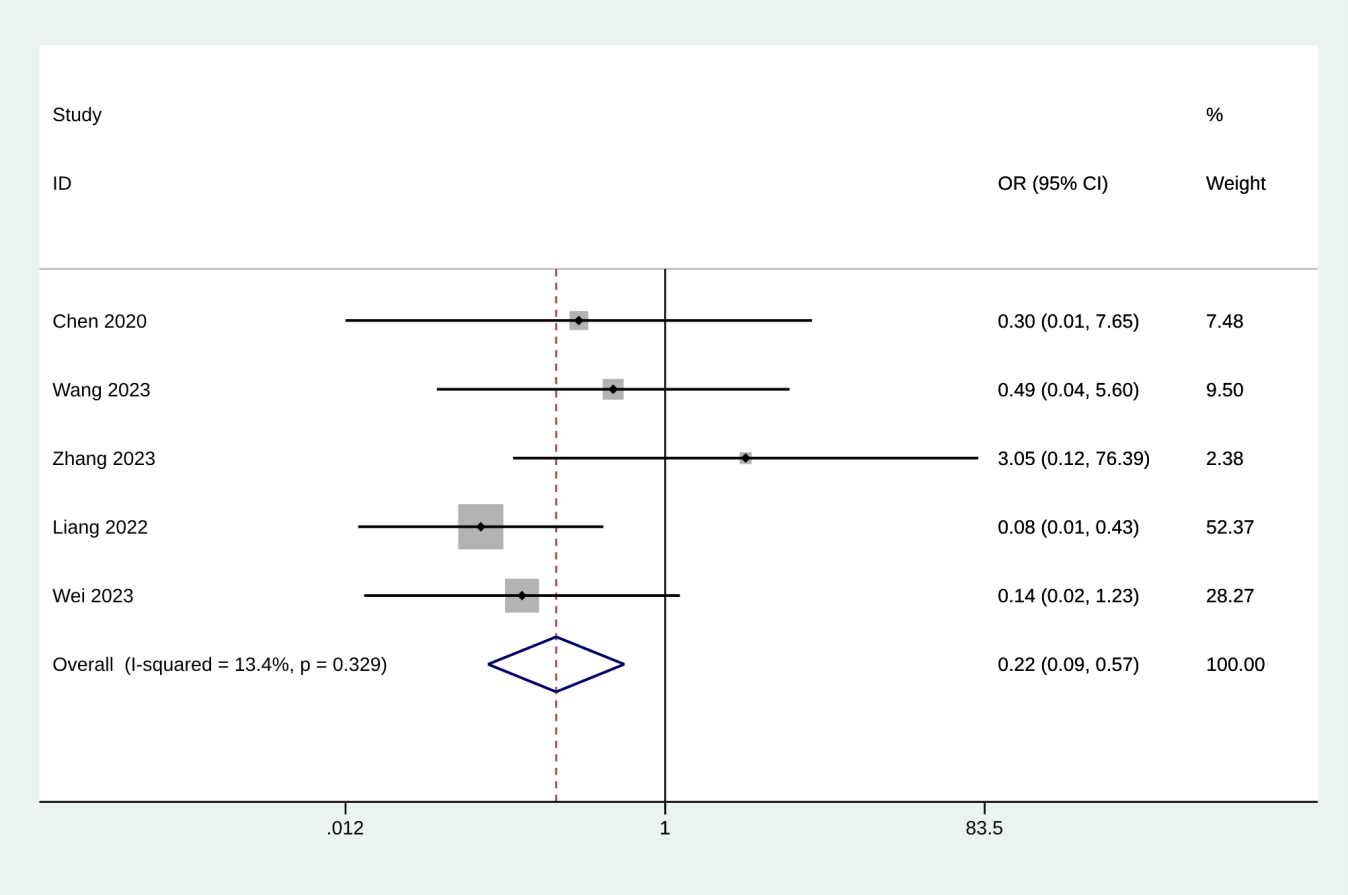
The effect of Baduanjin exercise on the adverse events. CI = confidence interval, OR = odds ratio.

Five studies reported the effect of Baduanjin exercise on adverse events. The meta-analysis results showed that Baduanjin exercise could prevent the adverse event from happening (odds ratio = 0.22, 95% CI 0.09 to 0.57, *P* = .002, *I*^2^ = 13.4%, Fig. 3). No significant publication bias was shown in the funnel plot (Fig. S9, Supplemental Digital Content, https://links.lww.com/MD/R61) and Egger test results (*P* = .647). The sensitivity analysis confirmed the stability of the results (Fig. S10, Supplemental Digital Content, https://links.lww.com/MD/R61).

#### 3.4.5. Left ventricular ejection fraction

**Figure 4.**
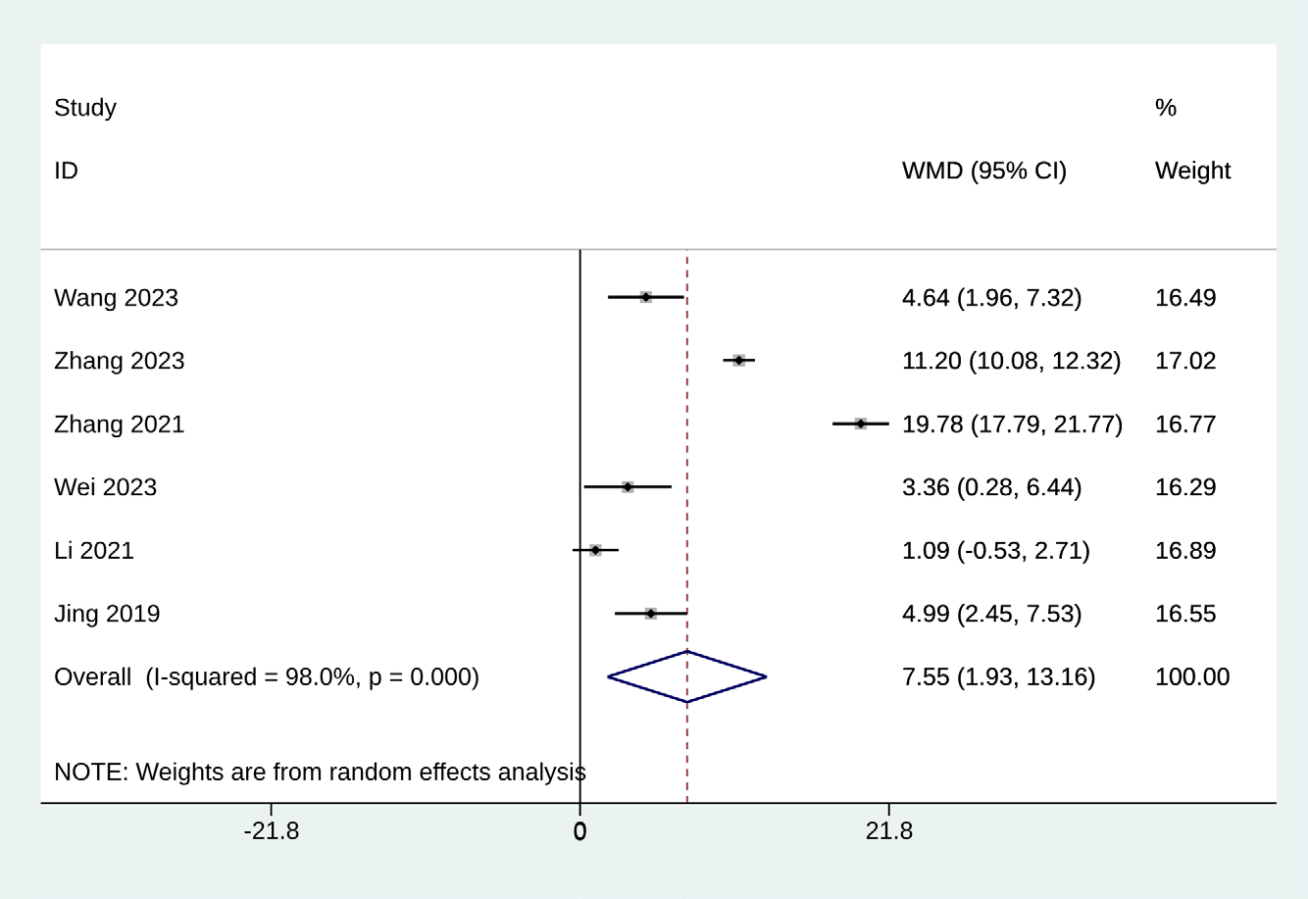
The effect of Baduanjin exercise on LVEF. CI = confidence interval, LVEF = left ventricular ejection fraction.

Six studies assessed cardiac function by LVEF in patients with or without Baduanjin exercise. Significant heterogeneity was observed (*P* < .001, *I*^2^ = 98%), so a random effect model was applied. Baduanjin exercise significantly improved LVEF compared with controls (MD = 7.55; 95% CI: 1.93 to 13.16; *P* < .001, Fig. 4). No publication bias was detected in the funnel plot (Fig. S11, Supplemental Digital Content, https://links.lww.com/MD/R61) and Egger test results (*P* = .592). The sensitivity analysis confirmed the stability of the results (Fig. S12, Supplemental Digital Content, https://links.lww.com/MD/R61).

## 4. Discussion

In this meta-analysis, we investigated the effects of Baduanjin exercise on the quality of life and cardiac function in patients with CHD after cardiac surgery. This review included 15 studies comprising 1384 participants. The pooled results indicated that Baduanjin exercise enhances quality of life measured by both the SF-36 and SAQ scales, reduces the occurrence of adverse events, and contributes to better cardiac function. These results indicated the safety and clinical usefulness of Baduanjin exercise for patients with CHD after cardiac surgery. This topic carries substantial public health significance, as the impact of Baduanjin on postoperative quality of life and cardiac function in individuals with cardiovascular disease has not been comprehensively evaluated.

Although previous studies have shown that patients with CHD can benefit from exercise after cardiac surgery, the magnitude of benefit may differ depending on the content, frequency, duration, and intensity of the specific exercise prescribed.^[18]^ Baduanjin, a traditional form of Chinese Qigong, features gentle, slow, and low-intensity movements that integrate physical postures, controlled breathing, and mindful regulation.^[39]^ Moreover, Baduanjin is simple and easy to learn and does not rely on fitness equipment. Patients with CHD after cardiac surgery often feel fatigued and find exercise exhausting, which may increase their risk of negative psychosocial outcomes, such as depression, anxiety, and social isolation. In this context, Baduanjin aligns well with the physical condition of patients with poor exercise tolerance and can serve as a primary form of rehabilitation exercise.

In addition, Baduanjin exercise has been reported to increase antioxidant enzymes, promote metabolism and blood circulation, lessen oxidative stress, and improve the negative mood associated with CHD, thereby contributing to improved life quality and cardiac function.^[11,40,41]^ Furthermore, existing evidence suggests that Baduanjin also has antidepressant and anxiolytic benefits.^[42]^ A possible explanation for the improvements in exercise capacity is that Baduanjin exercise helps patients mobilize their movable joints and muscles, improve balance, and enhance muscular strength.^[11]^

Previous meta-analyses have indicated that Baduanjin is a safe and feasible home-based rehabilitation method that enhances activity tolerance, lung function, and quality of life while reducing negative emotions after lung surgery.^[43,44]^ Regular practice has been reported to strengthen muscles and bones, improve circulation, and reduce blood pressure, lipids, and glucose, thereby promoting physical and mental health in patients after PCI.^[45]^ Another meta-analysis further indicated that the Baduanjin group was superior to the conventional western medicine treatment group in physical functioning, role-physical, general health, role-emotional, mental health, anxiety, LVEF, blood pressure, and stroke volume in patients with CHD.^[46]^ In this meta-analysis, it is shown that Baduanjin exercise significantly improved quality of life (SF-36 and SAQ), enhanced cardiac function (LVEF), and lowered adverse event incidence.^[46]^ These findings align with earlier research exploring the effects of Baduanjin exercise on the physical function, daily living activities and quality of life. By focusing on patients with coronary heart disease after cardiac surgery, our study expands the evidence base to a new patient population, further supporting the role of Baduanjin in improving cardiac function, regulating psychological state, and enhancing quality of life.

However, several limitations should be noted. First, most included articles were observational studies, which may introduce inherent bias. Second, the search was limited to Chinese and English databases. Given that Baduanjin originates from traditional Chinese medicine, all included studies were conducted in China, which may introduce language-related bias. Third, the intervention programs differed among studies in duration, frequency, and assessment timing, potentially contributing to the heterogeneity of the pooled findings. In addition, heterogeneity may also have been influenced by differences in participant characteristics, concomitant medications, and baseline disease status. To address this, we applied random-effects models and subgroup analyses, but residual heterogeneity may still remain. Lastly, the number of the included studies was relatively small. Therefore, the results of the present study should be interpreted with caution.

## 5. Conclusions

Our research results indicated that Baduanjin could improve the quality of life and cardiac function in CHD patients after cardiac surgery. However, more studies with rigorous research designs are needed to assist in the rehabilitation of such patients.

## Author contributions

**Conceptualization:** Xuezhi Liu, Xingxing Hao.

**Data curation:** Xuezhi Liu, Xingxing Hao, Wenhui Zhang.

**Formal analysis:** Xuezhi Liu.

**Writing – original draft:** Xuezhi Liu, Xingxing Hao, Wenhui Zhang, Fan Zhang, Hui Liu.

**Writing – review & editing:** Xuezhi Liu, Xingxing Hao, Wenhui Zhang, Fan Zhang, Hui Liu.

## Supplementary Material



## References

[1] Yang Y Wang B Cui J. Design and realization of MEMS heart sound sensor with concave, racket-shaped cilium. Biosensors (Basel). 2022;12:534. 35884337 10.3390/bios12070534 PMC9312695

[2] Weiss-Faratci N Lurie I Benyamini Y Cohen G Goldbourt U Gerber Y. Optimism during hospitalization for first acute myocardial infarction and long-term mortality risk: a prospective cohort study. Mayo Clin Proc. 2017;92:49–56. 27876316 10.1016/j.mayocp.2016.09.014

[3] Flygare O Boberg J Rück C. Association of anxiety or depression with risk of recurrent cardiovascular events and death after myocardial infarction: a nationwide registry study. Int J Cardiol. 2023;381:120–7. 37080468 10.1016/j.ijcard.2023.04.023

[4] Koivunen M Tynkkynen J Oksala N Eskola M Hernesniemi J. Incidence of sudden cardiac arrest and sudden cardiac death after unstable angina pectoris and myocardial infarction. Am Heart J. 2023;257:9–19. 36384178 10.1016/j.ahj.2022.11.009

[5] Subih M Elshatarat RA Sawalha MA. Exploring the impact of cardiac rehabilitation programs on health-related quality of life and physiological outcomes in patients post coronary artery bypass grafts: a systematic review. Rev Cardiovasc Med. 2024;25:145. 39076573 10.31083/j.rcm2504145 PMC11264007

[6] Johansson S Rosengren A Young K Jennings E. Mortality and morbidity trends after the first year in survivors of acute myocardial infarction: a systematic review. BMC Cardiovasc Disord. 2017;17:53. 28173750 10.1186/s12872-017-0482-9 PMC5297173

[7] Chen MG Liang X Kong L. Effect of Baduanjin sequential therapy on the quality of life and cardiac function in patients with AMI after PCI: a randomized controlled trial. Evid Based Complement Alternat Med. 2020;2020:8171549. 32714423 10.1155/2020/8171549 PMC7355341

[8] Chen J Zhou R Feng Y Cheng L. Molecular mechanisms of exercise contributing to tissue regeneration. Signal Transduct Target Ther. 2022;7:383. 36446784 10.1038/s41392-022-01233-2 PMC9709153

[9] Daniela M Catalina L Ilie O Paula M Daniel-Andrei I Ioana B. Effects of exercise training on the autonomic nervous system with a focus on anti-inflammatory and antioxidants effects. Antioxidants. 2022;11:350. 35204231 10.3390/antiox11020350 PMC8868289

[10] Saeidifard F Wang Y Medina-Inojosa JR Squires RW Huang H-H Thomas RJ. Multicomponent cardiac rehabilitation and cardiovascular outcomes in patients with stable angina: a systematic review and meta-analysis. Mayo Clinic Proc Innov Quality Outcomes. 2021;5:727–41. 10.1016/j.mayocpiqo.2021.06.009 PMC8325103 34355130

[11] Yang WY Xu Y Ye L. Effects of Baduanjin exercise on quality-of-life and exercise capacity in patients with heart failure: a systematic review and meta-analysis. Complement Ther Clin Pract. 2023;50:101675. 36436262 10.1016/j.ctcp.2022.101675

[12] Liu X Seah JWT Pang BWJ. A single-arm feasibility study of community-delivered Baduanjin (Qigong practice of the eight Brocades) training for frail older adults. Pilot Feasibility Stud. 2020;6:105. 32699644 10.1186/s40814-020-00649-3 PMC7372818

[13] Wu Z Hu Z Ke S. Multiform-based Baduanjin exercise prevention and treatment for idiopathic pulmonary fibrosis: study protocol for a randomized controlled trial. BMC Complement Med Ther. 2023;23:155. 37173702 10.1186/s12906-023-03974-1 PMC10177735

[14] Lu Y Qu HQ Chen FY. Effect of Baduanjin Qigong exercise on cancer-related fatigue in patients with colorectal cancer undergoing chemotherapy: a randomized controlled trial. Oncol Res Treat. 2019;42:431–9. 31266043 10.1159/000501127 PMC6771138

[15] Luo X Zhao M Zhang Y Zhang Y. Effects of Baduanjin exercise on blood glucose, depression and anxiety among patients with type II diabetes and emotional disorders: a meta-analysis. Complement Ther Clin Pract. 2023;50:101702. 36423358 10.1016/j.ctcp.2022.101702

[16] Konya P Demirtürk N Korkmaz D Tünay H Koşar EB. Evaluation of clinical and laboratory characteristics and factors affecting mortality in 500 hospitalized COVID-19 patients: a retrospective study. Saudi Med J. 2022;43:1254–9. 36379528 10.15537/smj.2022.43.11.20220641 PMC10043905

[17] Li X Lin Q Liu R Wu Y Fan Z. Role of Baduanjin exercise-based cardiac rehabilitation in coronary heart disease after percutaneous coronary intervention: a protocol for systematic review and meta-analysis of randomized controlled trials. Medicine (Baltim). 2022;101:e31612. 10.1097/MD.0000000000031612 PMC9771325 36550812

[18] Li L Zhang J Qiao Q Wu L Chen L. Development, reliability, and validity of the “Knowledge-Attitude-Practice” questionnaire of foreigners on traditional Chinese medicine treatment. Evid Based Complement Alternat Med. 2020;2020:8527320. 33144871 10.1155/2020/8527320 PMC7596425

[19] Wang X Yin X Liu P. The effect of Baduanjin Qigong combined with five-elements music on anxiety and quality of sleep in asymptomatic patients with COVID-19 infection: a randomised controlled trial. Heliyon. 2023;9:e18962. 37636423 10.1016/j.heliyon.2023.e18962 PMC10447985

[20] Page MJ McKenzie JE Bossuyt PM. The PRISMA 2020 statement: an updated guideline for reporting systematic reviews. BMJ. 2021;372:n71. 33782057 10.1136/bmj.n71 PMC8005924

[21] O’Brien KK Solomon P Bergin C. Reliability and validity of a new HIV-specific questionnaire with adults living with HIV in Canada and Ireland: the HIV Disability Questionnaire (HDQ). Health Qual Life Outcomes. 2015;13:124. 26263898 10.1186/s12955-015-0310-9 PMC4542093

[22] Goldsmith KA Dyer MT Buxton MJ Sharples LD. Mapping of the EQ-5D index from clinical outcome measures and demographic variables in patients with coronary heart disease. Health Qual Life Outcomes. 2010;8:54. 20525323 10.1186/1477-7525-8-54 PMC2900231

[23] Sterne JA Hernán MA Reeves BC. ROBINS-I: a tool for assessing risk of bias in non-randomised studies of interventions. BMJ. 2016;355:i4919. 27733354 10.1136/bmj.i4919 PMC5062054

[24] Sterne JAC Savović J Page MJ. RoB 2: a revised tool for assessing risk of bias in randomised trials. BMJ. 2019;366:l4898. 31462531 10.1136/bmj.l4898

[25] Yan W Shuqin P. The effect of modified Baduanjin on cardiopulmonary exercise capacity of patients with acute coronary syndrome after stent placement. China Med Guide. 2020;18:178–9.

[26] Wang X Liang Z Wenbao H. The effects of Baduanjin on negative emotions and quality of life in patients with coronary heart disease after PCI for phase II cardiac rehabilitation basic medical forum. MiNerVa Surg. 2023;27:4–7.

[27] Gong L Ming L Zhengbin S. Clinical observation on the intervention of Ba Duan Jin for patients with coronary heart disease and type 2 diabetes after PCI surgery. J Tradit Chin Med Clin Sci. 2021;33:1181–5.

[28] Lin X Chen J Guangqing Z. The influence of Baduanjin exercise on the quality of life of patients after coronary artery bypass grafting. J Nurs. 2012;19:63–7.

[29] Zhang X He S Xiaqing L. Exploring the effect of Ba Duan Jin exercise therapy on cardiac rehabilitation of patients with acute myocardial infarction after PCI surgery. China Health Standard Manage. 2023;14:137–40.

[30] Wang X Ye L Xianjun Y. Application of Baduanjin exercise in cardiac rehabilitation of patients with coronary heart disease after PCI. Cardiovasc Dis Prev Knowledge. 2019;9:33–7.

[31] Liang X Zhang X Minggui C. The impact of multi-dimensional follow-up on the compliance and quality of life of patients with acute myocardial infarction after PCI using the eight-stage qigong sequential therapy. J Pract Cardiopul Vasc Dis. 2022;30:85–90.

[32] Zhang Y Rao Y Yinghua L. Observation on the effect of nursing intervention based on “Seated Baduanjin” on promoting cardiac rehabilitation of patients after PCI. J Jiangxi Univ Chin Med. 2021;33:44–7.

[33] Gu F Wang P Chenglong W. Evaluation of the impact of Badong Qigong on the quality of life of patients with coronary heart disease after interventional therapy based on the Seattle Angina Questionnaire. J Integr Tradit West Medi Heart Cerebral Vasc Dis. 2018;16:2281–3.

[34] Wei N Meng Z. The effect of applying phase I cardiac rehabilitation combined with standing eight-section qigong nursing on patients with acute myocardial infarction after PCI treatment. Modern Med Health. 2023;39:1412–5.

[35] Chaolan R. Analysis of the impact of Baduanjin on the rehabilitation nursing of patients undergoing coronary heart disease intervention therapy. Contemp Clin Med J. 2022;35:85–6.

[36] Li S Yu M Xiang G. The effect of seated Ba Jin Qigong on the initial cardiopulmonary function of patients after coronary artery bypass grafting. J Integr Tradit West Med Cardiovasc Cerebral Dis. 2021;19:2879–84.

[37] Wang J Li R Lingyan Y. Application of seated Ba Jin Qigong exercise in the rehabilitation nursing of patients after PCI for coronary heart disease harbin pharmacy. Cardiovasc Dis Prevent Know. 2019;39:465–7.

[38] Chen T Zhang X Qingyuan S. Analysis of the therapeutic effect of Baduanjin on depressive state after coronary heart disease stent surgery. Med Diet Ther Health. 2021;19:9–11.

[39] Shao BY Zhang XT Vernooij RWM. The effectiveness of Baduanjin exercise for hypertension: a systematic review and meta-analysis of randomized controlled trials. BMC Complement Med Ther. 2020;20:304. 33032580 10.1186/s12906-020-03098-w PMC7545896

[40] Mao S Zhang X Shao B. Baduanjin exercise prevents post-myocardial infarction left ventricular remodeling (BE-PREMIER trial): design and rationale of a pragmatic randomized controlled trial. Cardiovasc Drugs Ther. 2016;30:315–22. 27106833 10.1007/s10557-016-6660-7

[41] Khan AA Al-Omary MS Collins NJ Attia J Boyle A. Natural history and prognostic implications of left ventricular end-diastolic pressure in reperfused ST-segment elevation myocardial infarction: an analysis of the thrombolysis in myocardial infarction (TIMI) II randomized controlled trial. BMC Cardiovasc Disord. 2021;21:243. 34001032 10.1186/s12872-021-02046-x PMC8130170

[42] Huang Y Xu X Chaurasiya BK Bizimana P Qian M-J Ntawuyamara E. Effects and safety of the traditional Chinese exercise Baduanjin on depression and anxiety in COVID-19 patients: a systematic review and meta-analysis. Complement Ther Med. 2024;86:103094. 39357550 10.1016/j.ctim.2024.103094

[43] Wu D Li J Dong H Zheng Y Cui H. The effect of Baduanjin on postoperative activity tolerance, lung function and negative emotions in patients with lung cancer: a systematic review and meta-analysis. Support Care Cancer. 2025;33:1–13. 10.1007/s00520-025-09690-5 40571840

[44] Wu D Li J Dong H Zheng Y Cui H. The effect of Baduanjin on postoperative activity tolerance, lung function and negative emotions in patients with lung cancer: a systematic review and meta-analysis. Support Care Cancer. 2025;33:631. 40571840 10.1007/s00520-025-09690-5

[45] Li X. The efficacy and safety of Baduanjin in patients with coronary heart disease after percutaneous coronary intervention: a systematic review and meta-analysis. 2022.

[46] Gai T-T Fan W Gao M-X Cui Y Wang Y. Effect of Baduanjin on rehabilitation of patients with coronary heart disease: a meta-analysis of 8 randomized controlled trials. Nurs Commun. 2019;3:133–9.

