# Supplementary material for: Baduanjin sequential therapy’s effects on quality of life and cardiac function in post-cardiac surgery heart disease patients: A systematic review
Source: Medicine (Baltimore). 2026 Jan 2;105(1):e46855. doi: 10.1097/MD.0000000000046855 (PMC12778147; doi:10.1097/MD.0000000000046855)
Supplement: Supplementary file 1 [file medi-105-e46855-s001.pdf]

Supplemental Digital Content Figure 1. Risk of bias summary for the studies included in the meta-analysis. "+": low risk of bias, "-": some concerns.

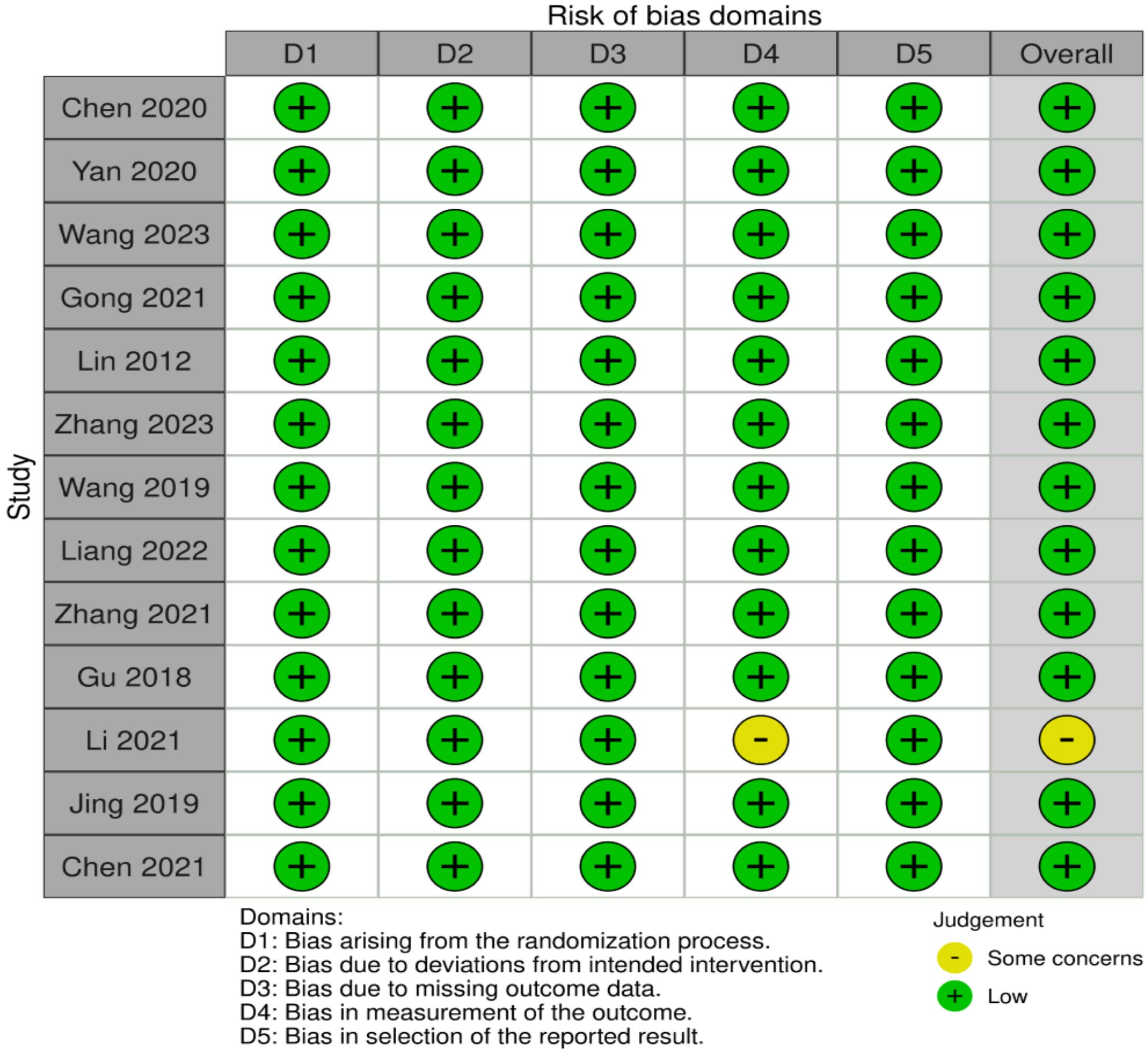

Supplemental Digital Content Figure 2. Risk of bias graph for the studies included in the meta-analysis.

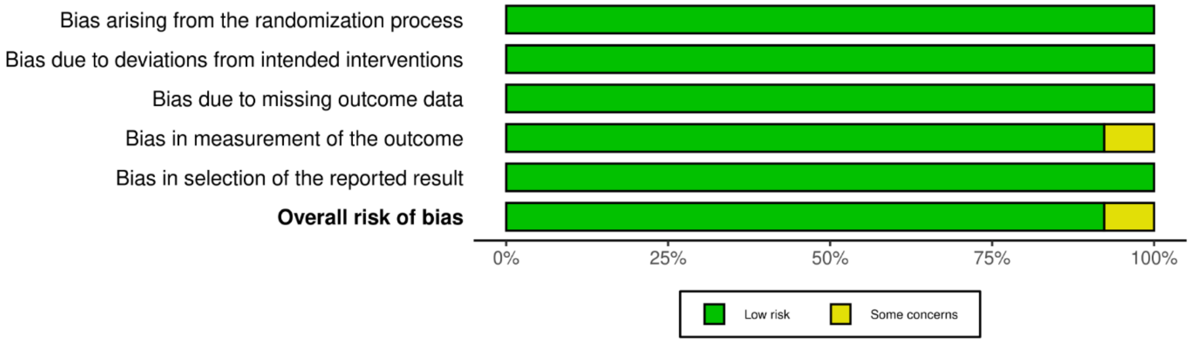

Supplemental Digital Content Figure 3. Funnel plot of the effects of Baduanjin on SF-36 sub-items. Bodily pain; (B) General health; (C) Mental health; (D) Physical functioning; (E) Role emotional; (F) Role physical; (G) Social function; (H) Vitality.

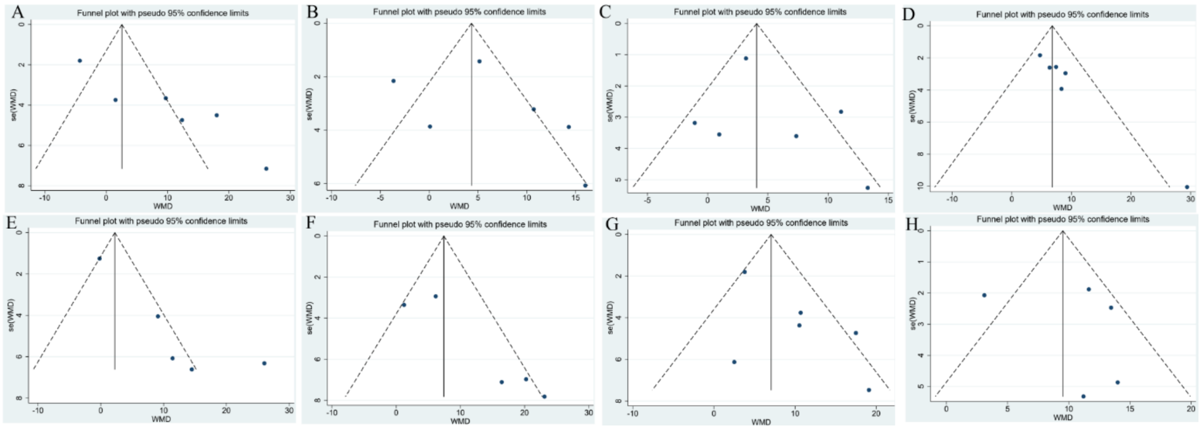

Supplemental Digital Content Figure 4. The sensitivity analysis of the effects of Baduanjin on SF-36 sub-items. Bodily pain; (B) General health; (C) Mental health; (D) Physical functioning; (E) Role emotional; (F) Role physical; (G) Social function; (H) Vitality.

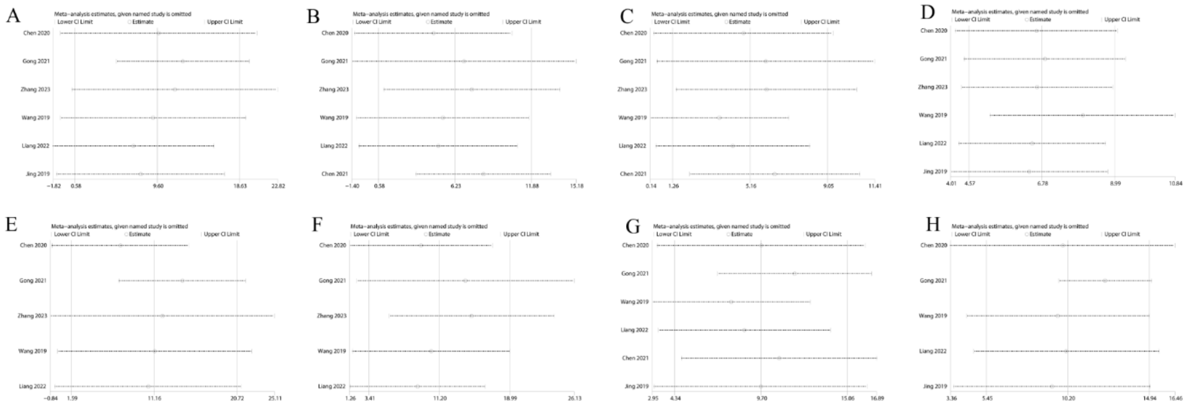

Supplemental Digital Content Figure 5. Funnel plot of the effects of Baduanjin on SAQ sub-items. Attack of angina pectoris; (B) Degree of disease awareness; (C) Physical limitation; (D) Stable angina pectoris; (E) Treatment satisfaction

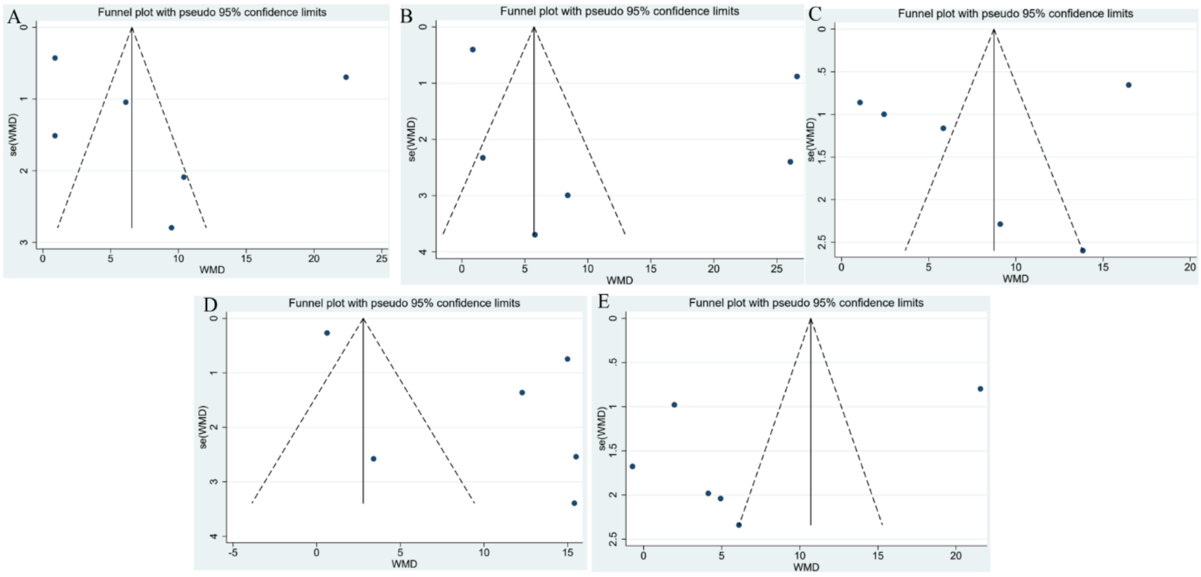

Supplemental Digital Content Figure 6. The sensitivity analysis of the effects of Baduanjin on SAQ sub-items. Attack of angina pectoris; (B) Degree of disease awareness; (C) Physical limitation; (D) Stable angina pectoris; (E) Treatment satisfaction

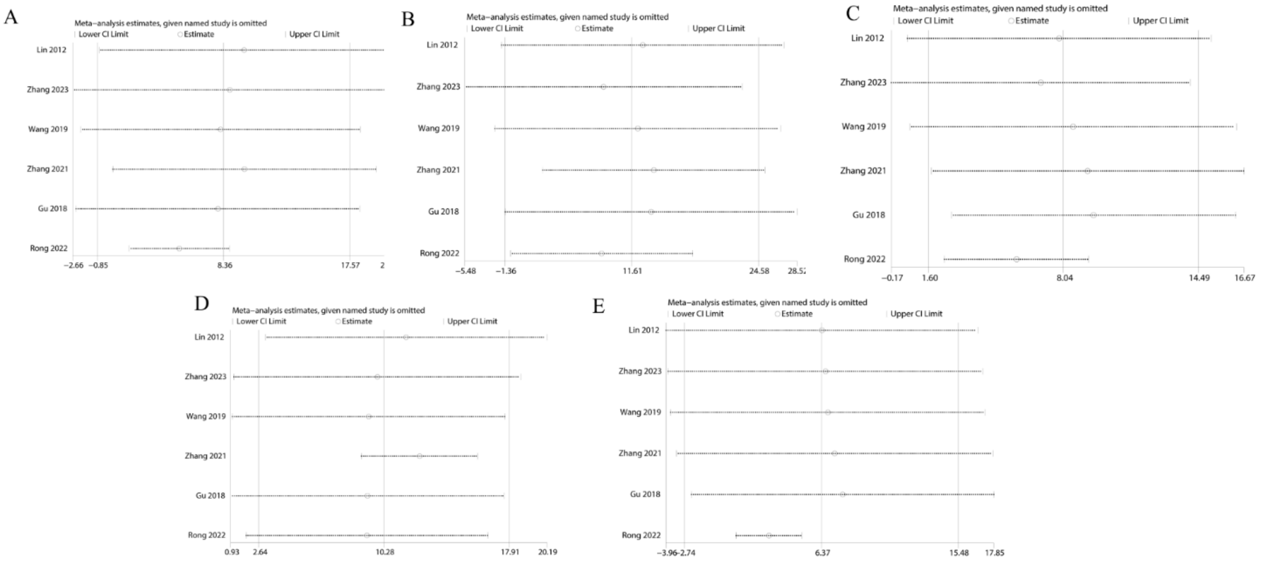

Supplemental Digital Content Figure 7. Funnel plot of the effects of Baduanjin on 6MWT.

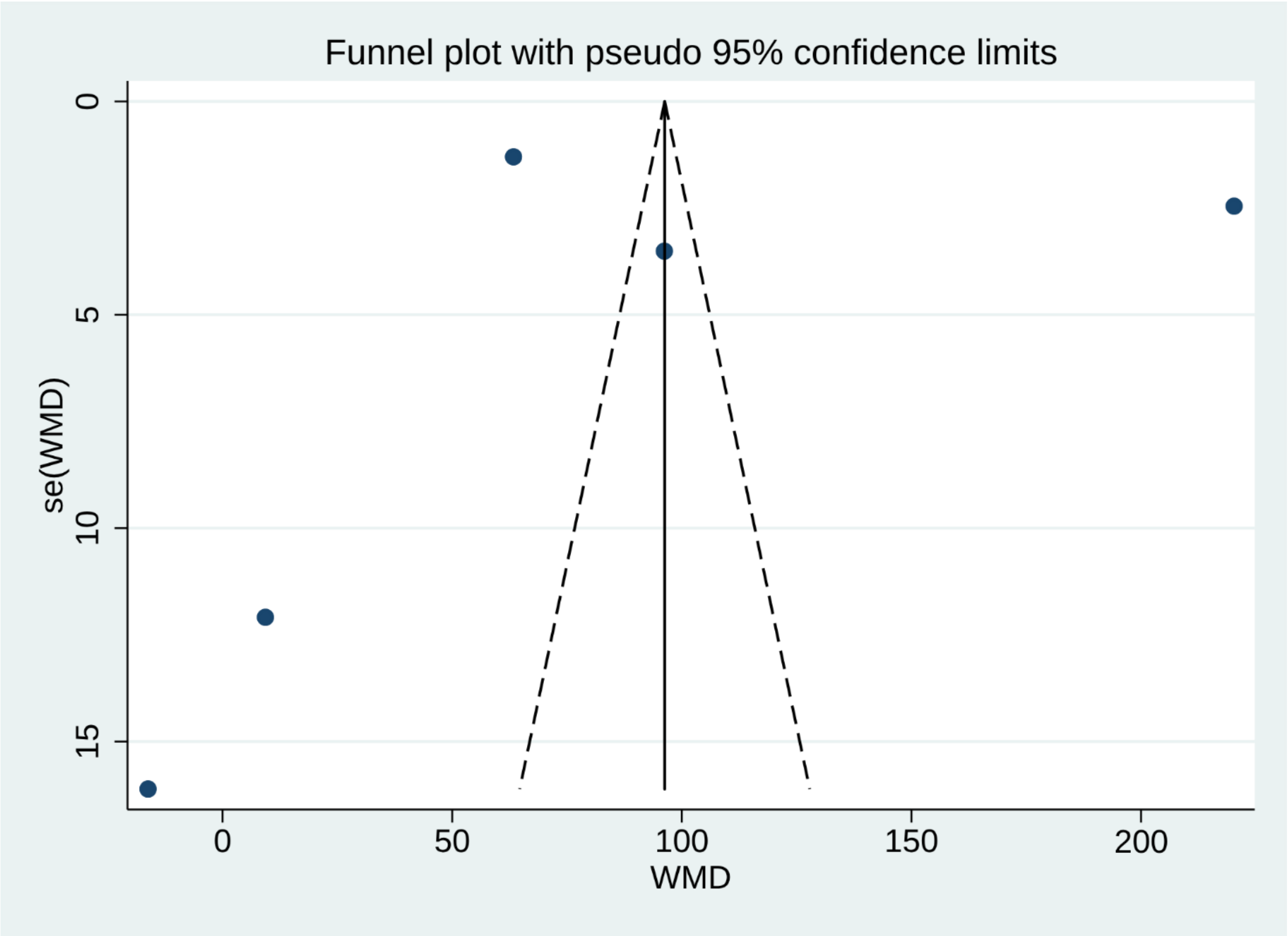

Supplemental Digital Content Figure 8. The sensitivity analysis of the effects of Baduanjin on 6MWT.

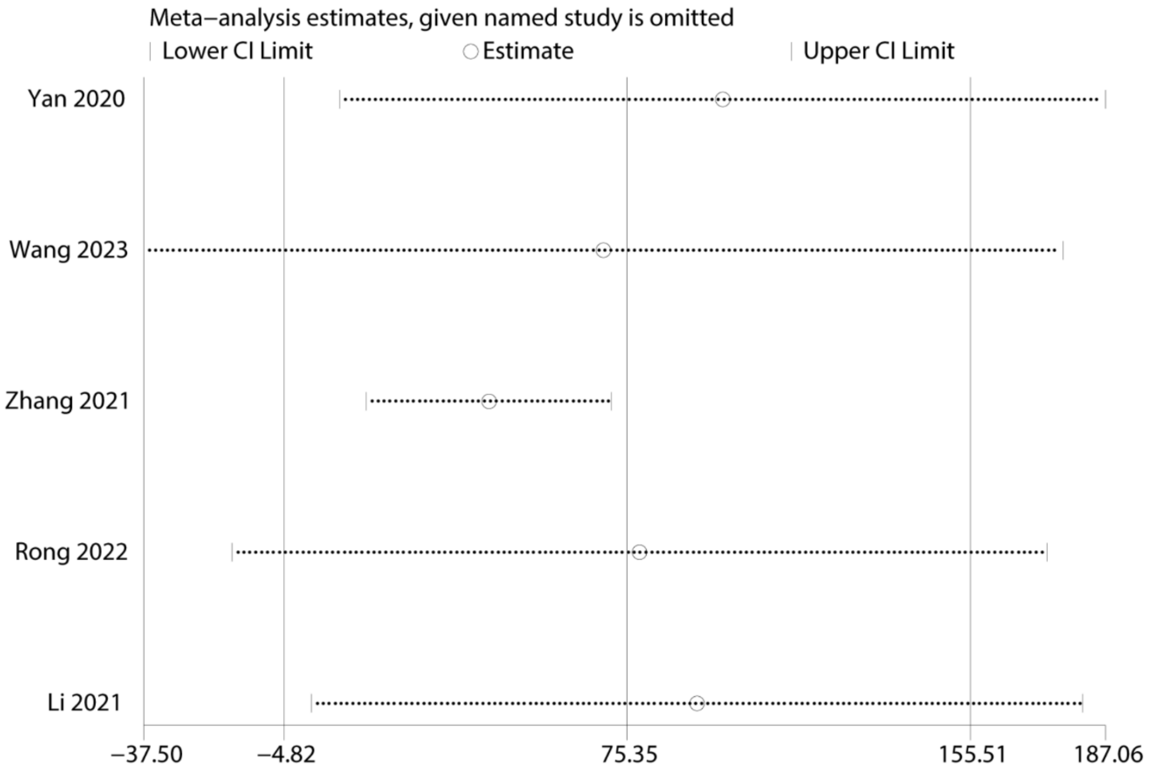

Supplemental Digital Content Figure 9. Funnel plot of the effects of Baduanjin on adverse events.

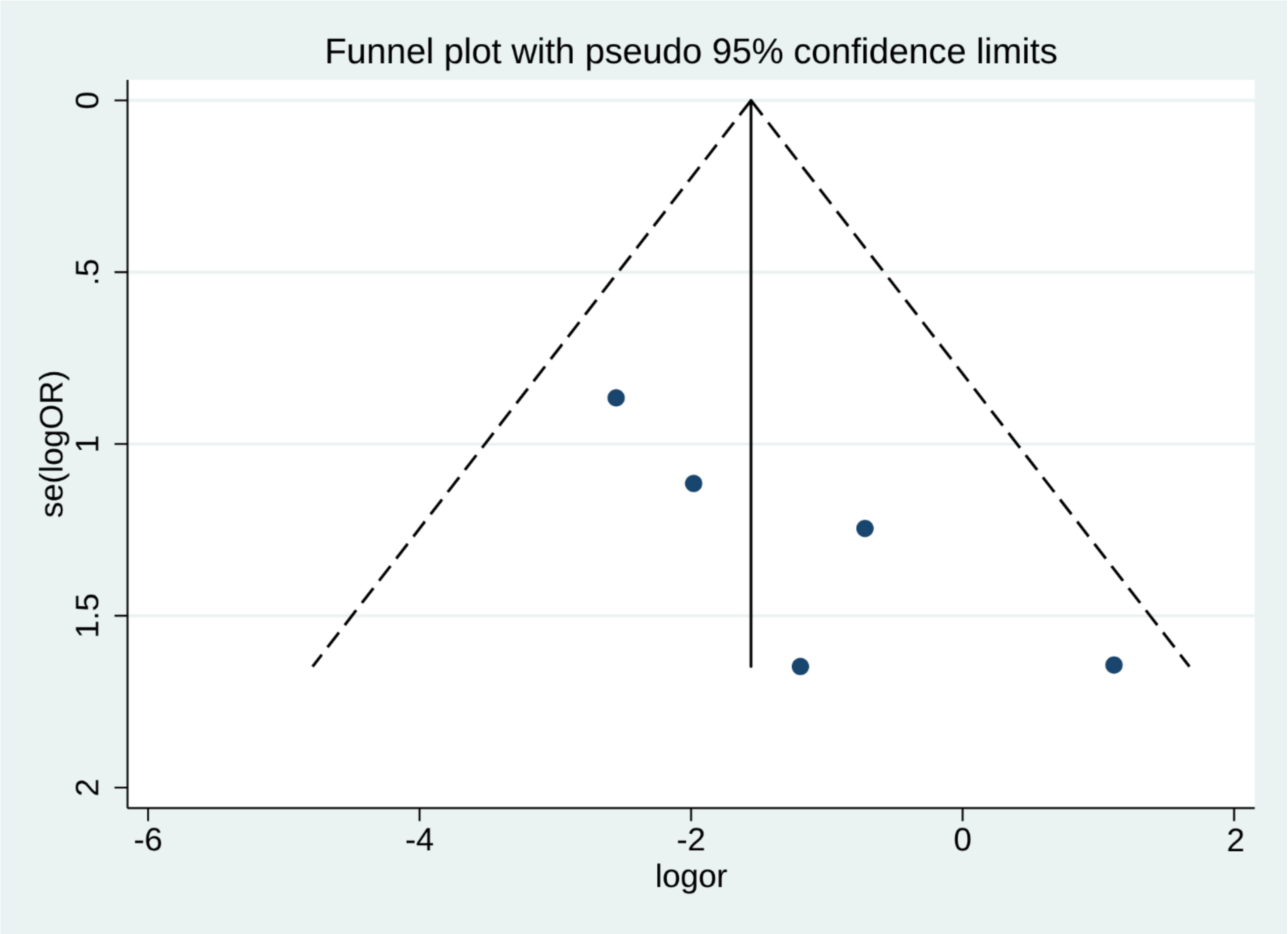

Supplemental Digital Content Figure 10. The sensitivity analysis of the effects of Baduanjin on adverse events.

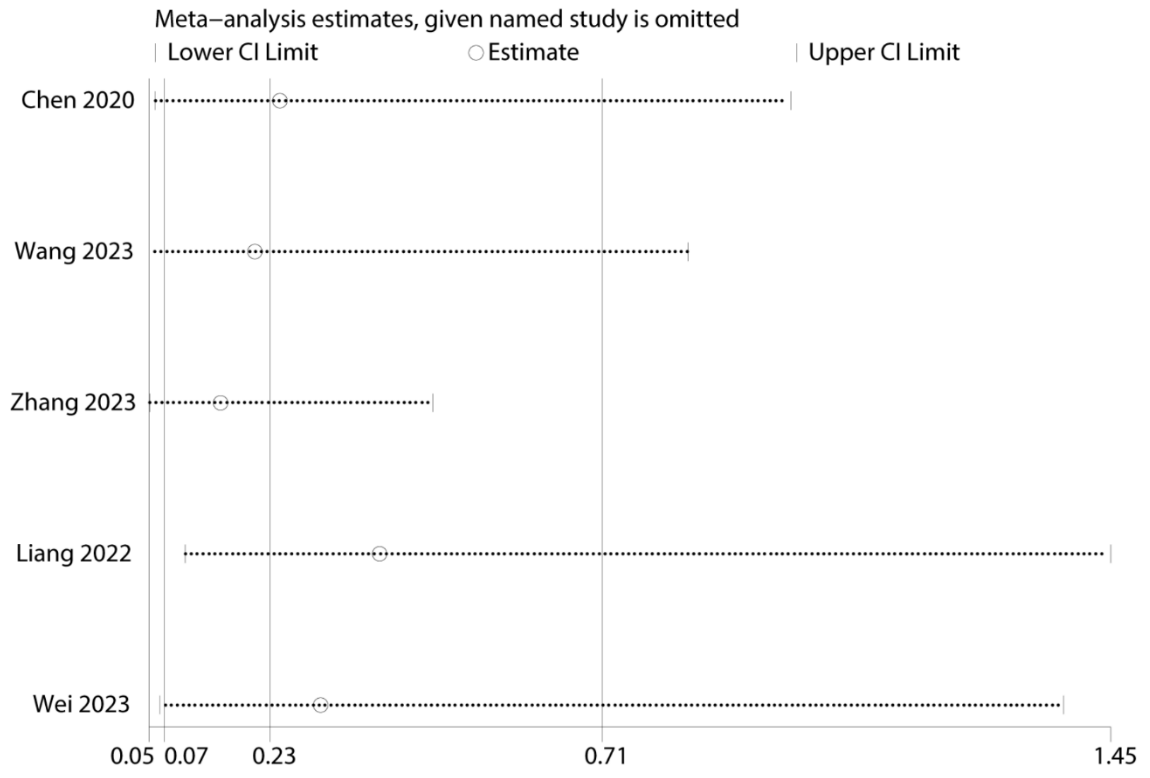

Supplemental Digital Content Figure 11. Funnel plot of the effects of Baduanjin on LVEF.

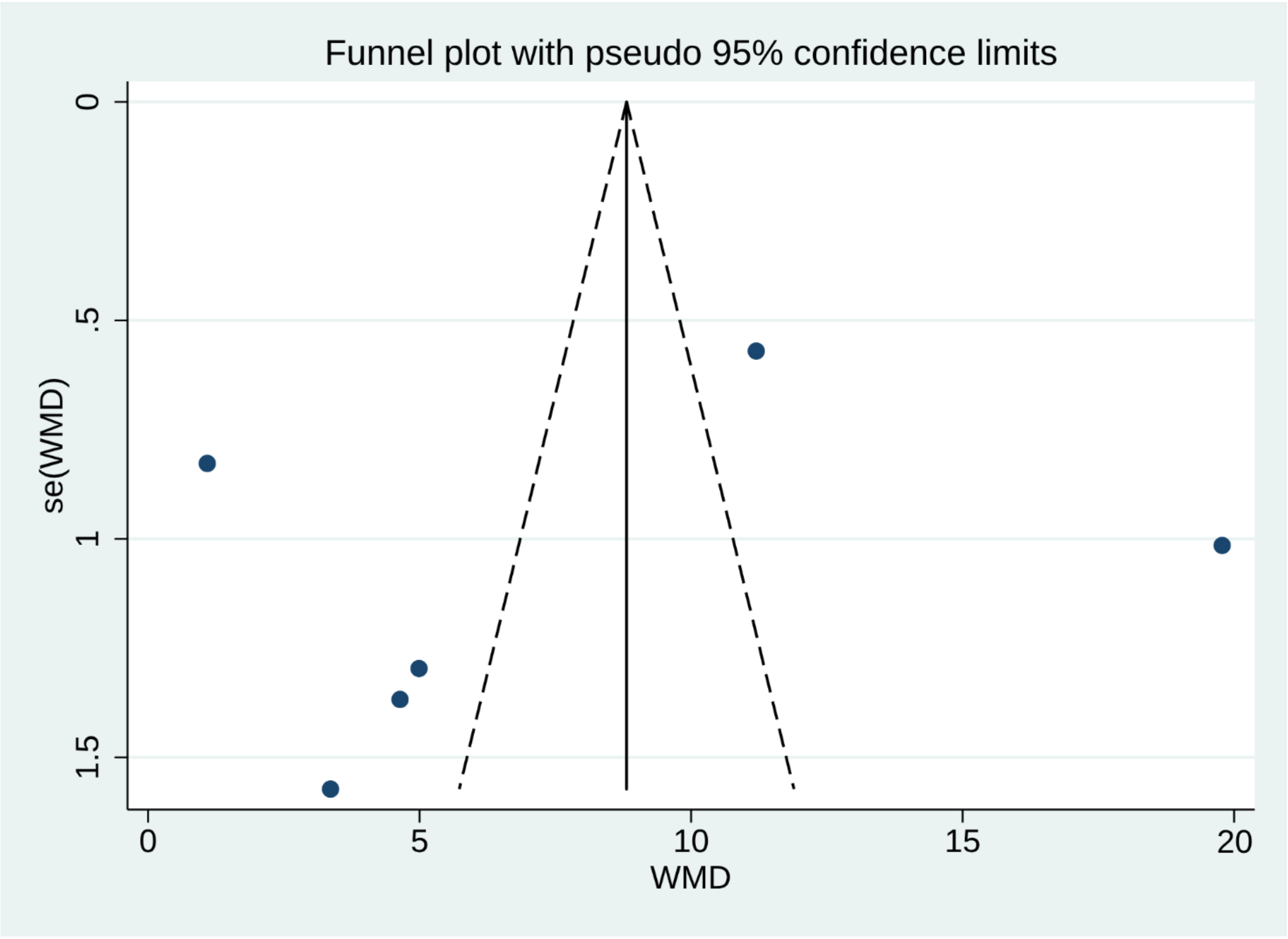

Supplemental Digital Content Figure 12. The sensitivity analysis of the effects of Baduanjin on LVEF.

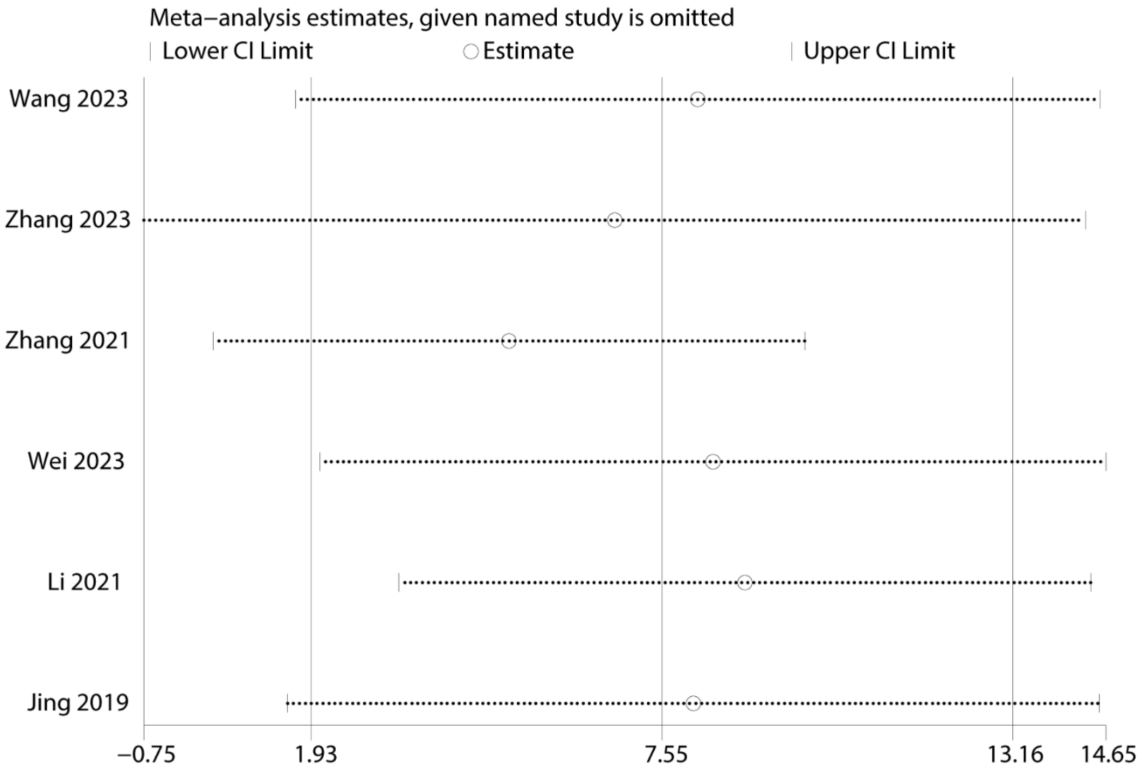

Supplemental Digital Content Table 1. Search strategy of PubMed

Search strategy of PubMed

NO.	Search Details	Results
#4	(#1 OR #2) AND #3	17
#3	((Baduanjin) OR (Ba Duan Jin Exercise)) OR (Qigong)	1,352
#2	(((((Coronary Diseases) OR (Coronary Heart Disease)) OR (Coronary Heart Diseases)) OR (coronary disease)) OR (multivessel coronary artery disease)) OR (coronary artery disease)	395,153
#1	"Coronary Disease"[Mesh]	239,118

Search strategy of EMBASE

No.	Query	Results
#4	#1 AND (#2 OR #3)	23
#3	'coronary diseases':ti,ab,kw OR 'coronary heart disease':ti,ab,kw OR 'coronary heart diseases':ti,ab,kw OR 'coronary disease':ti,ab,kw OR 'multivessel coronary artery disease':ti,ab,kw OR 'coronary artery disease':ti,ab,kw	269309
#2	'coronary artery disease'/exp	423110
#1	'baduanjin':ti,ab,kw OR 'ba duan jin exercise':ti,ab,kw OR 'qigong':ti,ab,kw	1724

Search strategy of Cochrane Library

NO.	Search deatiles	Hits
#1	MeSH descriptor: [Coronary Disease] explode all trees	18931
#2	(Coronary Diseases):ti,ab,kw OR (Coronary Heart Disease):ti,ab,kw OR (Coronary Heart Diseases):ti,ab,kw OR (coronary disease):ti,ab,kw OR (multivessel coronary artery disease):ti,ab,kw OR (coronary artery disease):ti,ab,kw	43191
#3	(Baduanjin):ti,ab,kw OR (Ba Duan Jin Exercise):ti,ab,kw OR (Qigong):ti,ab,kw	1019
#4	(#1 or #2) and #3	20

Search strategy of web of science

NO.	Search deatiles	Hits
#1	(TS=(Baduanjin) OR TS=(Ba Duan Jin Exercise)) OR TS=(Qigong)	1698
#2	((((TS=(Coronary Diseases) OR TS=(Coronary Heart Disease)) OR TS=(Coronary Heart Diseases)) OR TS=(coronary disease)) OR TS=(multivessel coronary artery disease)) OR TS=(coronary artery disease)	354496
#3	#2 AND #1	26

Search strategy of CNKI

(TKA = 'baduanjin' OR TKA = 'baduanjingongfa' OR TKA = 'baduanjinduannian' OR TKA = 'baduanjinganyu') AND (TKA = 'guanxinbing' OR TKA = 'guanxinbingjieru') 150

Search strategy of WanFang

(zhuti:(baduanjin) or zhuti:(baduanjingongfa) or zhuti:(baduanjinduannian) or zhuti:(baduanjinganyu)) and (zhuti:(guanxinbing) or zhuti:(guanxinbingjieru)) 150

Search strategy of Sinomed

("guanxinbing"[changyong ziduan:zhineng] OR "guanxinbingjieru"[changyong ziduan:zhineng]) AND ("baduanjin"[changyong ziduan:zhineng] OR "baduanjingongfa"[changyong ziduan:zhineng] OR "baduanjinduannian"[changyong ziduan:zhineng] OR "baduanjinganyu"[changyong ziduan:zhineng]) 108
